# Porphyrin Co(III)-Nitrene Radical Mediated Pathway for Synthesis of *o*-Aminoazobenzenes

**DOI:** 10.3390/molecules23051052

**Published:** 2018-05-01

**Authors:** Monalisa Goswami, Bas de Bruin

**Affiliations:** Homogeneous, Supramolecular and Bio-Inspired Catalysis, van ‘t Hoff Institute for Molecular Sciences, University of Amsterdam, Science Park 904, 1098 XH Amsterdam, The Netherlands; m.goswami@uva.nl

**Keywords:** azides, azobenzenes, nitrene radicals, base-metals, dyes, molecular switches

## Abstract

Azobenzenes are versatile compounds with a range of applications, including dyes and pigments, food additives, indicators, radical reaction initiators, molecular switches, etc. In this context, we report a general method for synthesizing *o*-aminoazobenzenes using the commercially available cobalt(II) tetraphenyl porphyrin **[Co^II^(TPP)]**. The net reaction is a formal dimerization of two phenyl azides with concomitant loss of two molecules of dinitrogen. The most commonly used methodology to synthesize azobenzenes is based on the initial diazotization of an aromatic primary amine at low temperatures, which then reacts with an electron rich aromatic nucleophile. As such, this limits the synthesis of azobenzenes with an amine functionality. In contrast, the method we report here relies heavily on the *o*-amine moiety and retains it in the product. The reaction is metal catalyzed and proceeds through a porphyrin Co(III)-nitrene radical intermediate, which is known to form on activation of organic azides at the cobalt center. The synthesized *o*-aminoazobenzenes are bathochromatically shifted, as compared to azobenzenes without amine substituents. Based on the crystal structure of one of the products, strong H-bonding between the N-atom of the azo functionality and the H of the NH_2_ substituent is shown to stabilize the *trans* isomeric form of the product. The NH_2_ substituents offers possibilities for further functionalization of the synthesized azo compounds.

## 1. Introduction

Azobenzenes are versatile compounds with a range of applications, including dyes and pigments, food additives, indicators, radical reaction initiators, and therapeutic agents [1,2,3,4,5,6,7,8,9,10]. In addition, azo compounds have shown promise in electronics and drug delivery [11]. They have also been proposed to be useful for applications in areas of nonlinear optics, chemosensors, liquid crystals, photochemical molecular switches, molecular shuttles, nanotubes, and in the manufacture of protective eye-glasses and filters [1,2,3,4,5,6,7,8,9,10]. To fully exploit the potential of these molecules for such versatile applications, it is important that they be easy to synthesize. A review on the synthesis of azobenzenes by Merino in 2011 summarizes all of the commonly employed methods for synthesizing azobenzenes [11]. The most commonly used method is based on the initial diazotization of an aromatic primary amine at low temperature, which then reacts with an electron rich aromatic nucleophile. As such, this limits the synthesis of azobenzenes, which have an amine functionality.

Ring substituents lead to drastic changes in the absorption, emission and photochemical properties of azobenzene [12]. Most of the azobenzene-modified biomolecules developed so far can undergo photoisomerization upon irradiation with UV light [13]. Azobenzene derivatives for which photoisomerization can occur entirely in the visible region are desirable for in vivo applications. A handful of examples exist in which introduction of suitable substituents makes the switching of these molecules possible at longer wavelengths, thereby obviating the need to use UV irradiation [14,15,16]. Therefore, catalytic methods, with functional group tolerance, for the synthesis of amine containing azo-compounds will aid in the further development of this field.

**Scheme 1:**
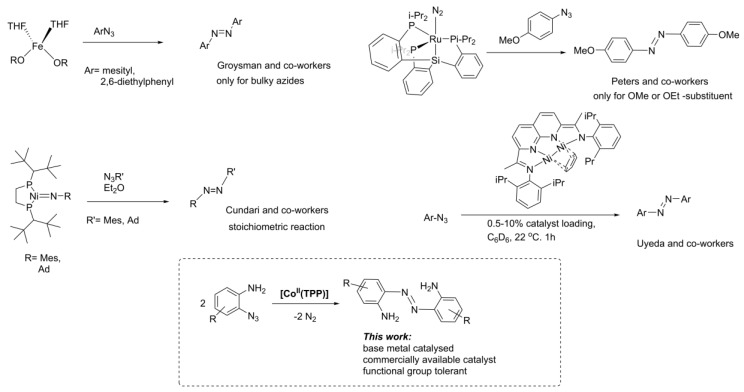
Summary of reported transition metal complexes for synthesis of azobenzenes and the catalytic reaction reported in this work.

To date, only a few examples of catalytic synthesis of azobenzenes via azides have been reported. These are summarized in Scheme 1. The iron-based example of Groysman and co-workers is limited in the sense that only azides with bulky substitutents like mesityl groups result in formation of azo compounds [17]. With trifluoromethyl and methyl substituents, dimers of the metal complex are obtained. The other example from Cundari and co-workers [18] involves a nickel complex, but this system produces only stoichiometric amounts of azo compounds. An example involving a ruthenium metallo-radical system proceeds via a free nitrene intermediate and works catalytically only for aryl azides with electron-rich substituents such as OMe and OEt [19]. Very recently, another dinuclear nickel complex was reported to be effective in the homo- and hetero-coupling of azides, giving azoarenes by means of the concomitant release of dinitrogen. The mononuclear version of the same complex did not lead to catalytic turnover [20].

As part of our previous publication in 2016, we reported a unique reaction that led to the synthesis of the simple *o*-aminoazobenzene in high yield using a cobalt(II) porphyrin catalyst [21]. In this regard, we now report a general catalytic synthesis of substituted *o*-aminoazobenzenes in two steps starting from commercially available substituted anilines. In this method, the anilines are first *o*-azidated using a reported Cu-catalyzed route [22]; then, in the unique cobalt(II) porphyrin-catalyzed pathway, the azides are activated by cobalt, leading to net ‘nitrene dimerization’, giving the azobenzenes. Cobalt(II) porphyrins have emerged as a new class of catalysts that can perform carbene and nitrene transfer reactions via discreet Co(III)-carbene or nitrene radicals [23,24,25,26]. The advantage of these cobalt(II) porphyrins lies in the fact that they can activate organic azides, thus obviating the need to use hypervalent iodine compounds like iminoiodanes or Halomine-Ts. Thus, cobalt(II) porphyrins are excellent catalysts for cleaner and milder access to nitrene transfer reactions. The utility of the method we report here is two-fold. Firstly, this catalytic method allows for a mild chemical method that is tolerant to primary amines to access azobenzenes via azides. The only by-product in this key step is dinitrogen. The only other way to access azobenzenes from organic azides is by uncatalyzed thermolysis, and the explosive nature of the azides is often pointed out as a disadvantage of using azides in such high-temperature uncatalyzed processes. Secondly, as *ortho* substituents are known to have dramatic effects in the photochemical properties of azobenzenes, this method gives access to a series of *o*-amino-substituted azobenzenes that have thus far not been extensively studied. The primary amine substituent can provide an easy handle for further functionalization. This presents new possibilities for the use of azobenzenes in a variety of applications including optical switches. Overall this method allows for synthesis of new azobenzenes starting from commercially available anilines in good to excellent isolated yields.

## 2. Results

**Scheme 2:**
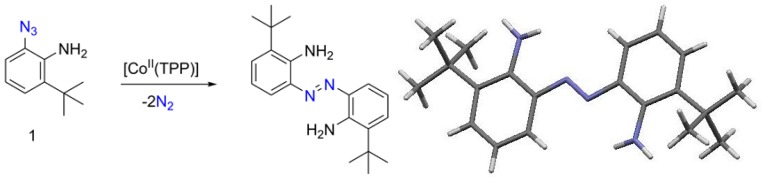
**Left: [Co^II^(TPP)]** catalyzed reaction of 2-azido-6-(*tert*-butyl)aniline, giving the corresponding trans-azobenzene. **Right:** Crystal structure of the thus-formed *o*-aminoazobenzene product.

As a test substrate **1** (2-azido-6-(*tert*-butyl)aniline) was synthesized according to the method described by Jiao and co-workers [22]. **1** (0.3 mmol) and **[Co^II^(TPP)]** (5 mol%) were dissolved in freshly distilled toluene and the reaction mixture was heated at 90 °C for 18 h (Scheme 2). During this time, the reaction proceeded cleanly, giving the corresponding azobenzene in near quantitative yield. The product was isolated by running a preparatory thin layer chromatography (prep-TLC) in pure dichloromethane (DCM). The isolated compound was a deep red-colored solid and was crystallized to confirm the formation of the *o*-aminoazobenzene product. 

With these results in hand, we set out to optimize this reaction further. Unfortunately, lowering the catalyst loading and/or temperature was detrimental to the reaction. These results are summarized in Table 1. The reaction temperature plays a very important role in this reaction. With 1 mol% catalyst loading in toluene at 90 °C the reaction proceeded, but the yields dropped (entry 4). Lower temperatures didn’t lead to any azobenzene formation in benzene or in THF (entries 2 and 5). Without catalyst present, the azide was unreacted and could be fully recovered from the reaction mixture (entry 6).

**Figure 1:**
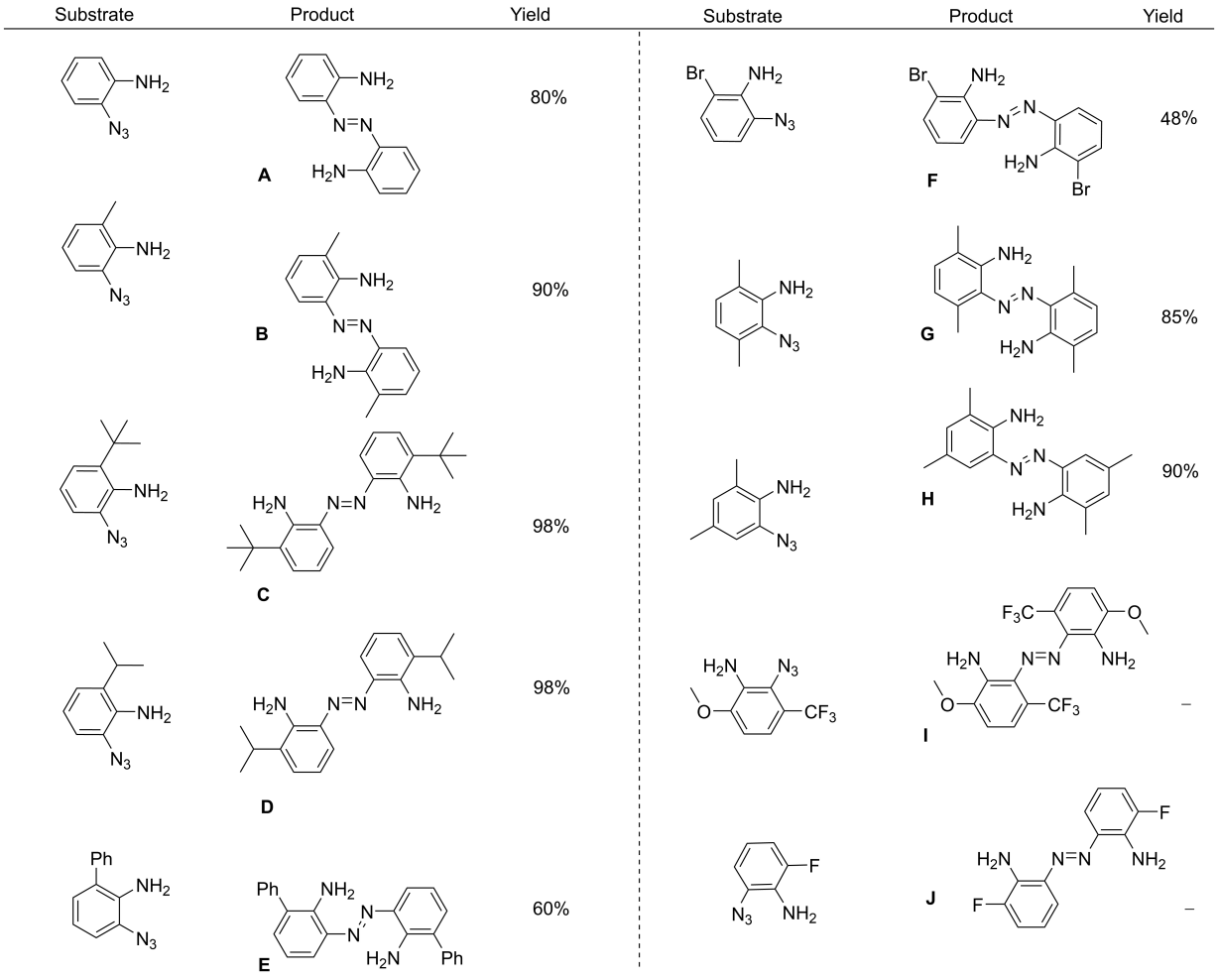
Substrate screening for **[Co^II^(TPP)]** catalyzed synthesis of azobenzenes from *o*-amine substituted azides *. * All reactions were carried out with 0.3 mmol of azide, **[Co^II^(TPP)]** (5 mol%) in 4 mL of toluene. The reaction mixtures were bubbled with dinitrogen for 15 min prior to thermostatting at the stated temperature for 18 h. Isolated yields are reported.

Using the optimized reaction conditions as determined above, we proceeded towards synthesizing various other substituted *o*-azidoanilines. The results are summarized in Figure 1. Substrates with electron-donating substituents performed better in this reaction than those with electron-withdrawing groups. For example, the phenyl substitution in **E** gave 60% of the product, while bromine substitution in **F** gave a 48% yield. Furthermore, in catalytic reactions using substrate **I** containing a CF_3_ substituent (and a MeO substituent) or substrate **J** containing a fluorine substituent, no azobenzene products were formed, and in some cases (entries **I** and **J**) starting azide was recovered. Apparently, with these azides, the cobalt is not able to activate the azides to give the crucial nitrene-radical intermediate. Substrates containing *tert*-butyl (**C**) or *iso*-propyl (**D**) groups gave excellent yields (see Supporting Information for spectra).

**Scheme 3:**
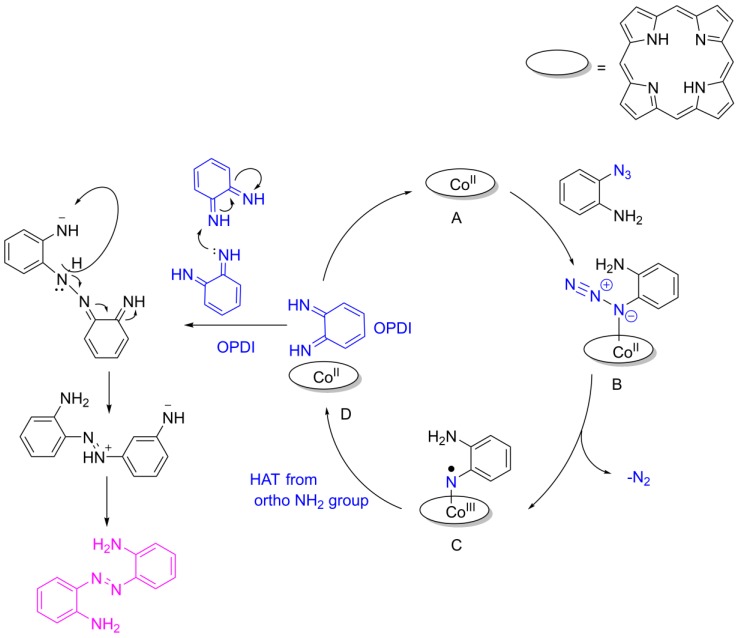
Proposed mechanism of the Co(II) porphyrin-catalyzed route to *o*-amino-azobenzenes via *o*-amino-azides.

As reported previously by us, the reaction is believed to proceed via the mechanism outlined in Scheme 3 [21]. The formation of azobenzene from *ortho*-amino phenyl azides is proposed to proceed via the phenylene diimine (**OPDI**) intermediate can be reasoned in the mechanism depicted in Scheme 3. Upon activation of the azide by the cobalt(II) porphyrin, a nitrene radical intermediate **C** is formed. Such nitrene radical intermediates have previously been fully characterized by us [25,26]. In the presence of an *ortho*-NH_2_ substituent, this nitrene radical intermediate undergoes an intramolecular H-atom abstraction from the amine substituent. This leads to formation of an **OPDI** intermediate, which couples to another **OPDI** molecule. Rearrangement of a proton then leads to the formation of the azobenzene product, as depicted in Scheme 3. The barrier for the intramolecular HAT step in going from **C** to **D** was previously reported by us and was found to be only +9.1 kcal mol^−1^; the overall process was exergonic by −3.1 kcal mol^−1^.

## 3. Electronic Properties of the Synthesized *o*-Amino-Substituted-Azobenzenes

**Figure 2:**
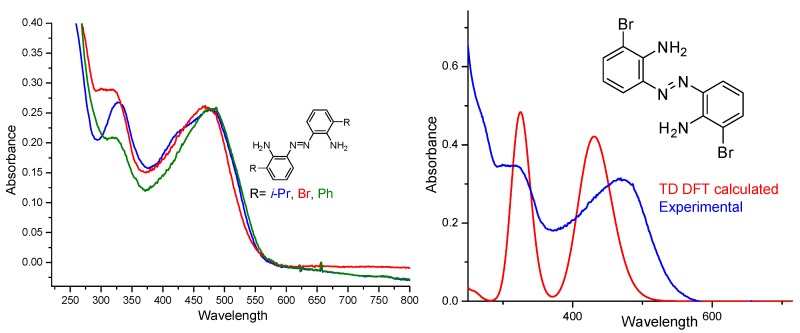
UV-vis spectra of o-aminoazobenzenes **D** (R = *i*-Pr), **E** (R = Ph) and **F** (R = Br) with varying electronic substituents in solvent acetonitrile (**left**). TD-DFT calculated (blue) and experimental UV-vis spectra (red) of compound **F** (**right**).

In addition to the synthesis, we also recorded the UV-vis spectra of the synthesized *o*-aminoazobenzenes to see what effect the amine substituent has on the absorption spectra. As expected, all the synthesized compound products showed a red shift of the π-π* and n-π* transitions in comparison to the parent azobenzene compound. Three such UV-vis spectra with electronically different substituents are shown in Figure 2 (left). The n-π* transitions are shifted to wavelengths above 450 nm, and π-π* transitions between 300 and 350 nm are of almost equal intensity to the n-π* transitions. For azobenzenes with electron-donating substituents, the π-π* transition is more red-shifted than for those with electron-withdrawing substituents (Figure 2, left). Time-dependent DFT (TD DFT) calculation reasonably reproduced these experimentally observed transitions and relative intensities. For example, for the bromo-substituted compound **F** the π-π* transition value matched almost exactly (λ = 323 nm), while the n-π* transition was more red shifted in reality than was predicted by the TD DFT calculations (λ = 430 nm (TD DFT) and 463 nm (experimental)).

**Figure 3:**
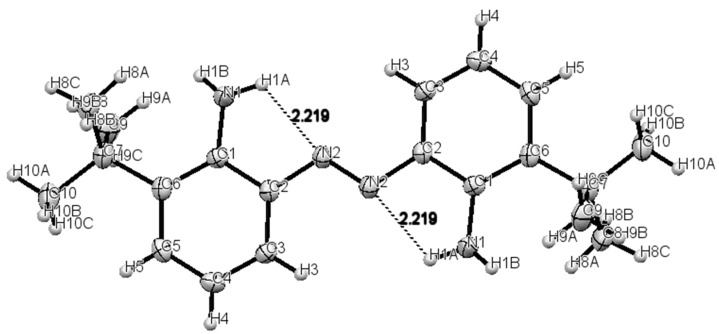
The NH⋯N=N hydrogen bonds in **C** as revealed by X-ray diffraction studies (CCDC 1828796).

H-bonding between the H atom of the NH_2_ and the N atom of the azo group was evident from the crystal structure of compound **C**. The NH⋯N=N hydrogen bond was found to be 2.219 Å. Such H-bonding interactions are known to hinder the isomerization pathway between the *trans*- and the *cis*- isomers of amino-azobenzenes (Figure 3). Additionally, in 2-hydroxy-azobenzenes, intramolecular H-bonding between the azo-nitrogen atom and the hydroxyl group is reported to lock the molecule in the *trans* conformation [27]. The 2-hydroxyazobenzenes provide a versatile platform for the design of reversible photoacids to generate significant pH pulses and oscillations with monochromatic light. Similar behavior can perhaps be expected for the *ortho*-amino-azobenzenes reported here, but this is beyond the scope of the current study.

## 4. Materials and Methods

All NMR spectra were recorded at 293 K. ^1^H-NMR: Bruker Avance 400 (400 MHz) (Rheinstetten, Germany or Varian Mercury 300 (300 MHz) (Palo Alto, California) was used, referenced internally to residual solvent resonance of CDCl_3_ (δ = 7.26 ppm). ^13^C{^1^H} NMR: Bruker Avance 400 (101 MHz) or Bruker Avance 500 (126 MHz), referenced internally to residual solvent resonance of CDCl_3_ (δ = 77.2 ppm). High-Resolution Mass spectra were measured on an AccuTOF LC, JMS-T100LP Mass spectrometer (JEOL, Tokyo, Japan), Needle voltage 2000 V, Orifice 1 voltage 90 V, Orifice 2 voltage 9 V, Ring Lens voltage 22 V. Ion source temperature 30 °C, solution flow rate 0.01 mL/min. All mass spectra were recorded with an average duration of 1 min. All chemicals were purchased from commercial sources unless otherwise mentioned. Solvents for all catalytic reactions were freshly distilled from sodium for toluene and for acetonitrile over calcium hydride. Reactions were performed using standard Schlenk techniques under an atmosphere of dinitrogen.

### 4.1. Synthesis of the Azides

**Caution:** All azides were synthesized in 1 mmol scale reactions in separate Schlenk tubes. After the reactions were complete, they were combined together before work-up and column separation.


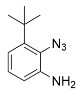


*2-azido-6-(tert-butyl)aniline*. 2-azido-6-(*tert*-butyl)aniline was synthesized according to the reported procedure of Jiao [22] and the spectral data matched with those reported [22].


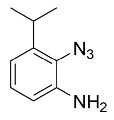


*2-azido-6-iso-propylaniline.* 1 mmol of isopropylaniline was added to a flame-dried Schlenk tube containing 0.1 mmol CuBr. Then trimethyl silyl azide (2 mmol) was added, followed by addition of 4 mL of freshly distilled acetonitrile. Finally, 2 mmol of tetrabutyl hydroperoxide (TBHP) (5.0–6.0 M in decane) was added, and the reaction was thermostatted at 30 °C for 6 h. After this, 15 mL of ethyl acetate was added the reaction mixture concentrated on a rotary evaporator. This was then directly loaded onto a silica column and eluted with pet ether:ethylacetate (60:1). ^1^H-NMR (400 MHz, Chloroform-*d*): δ 7.04–6.87 (m, 2H), 6.81 (t, *J* = 7.8 Hz, 1H), 3.84 (s, 3H), 2.98–2.66 (m, 1H), 1.25 (d, *J* = 6.8 Hz, 6H). IR: 2110 cm^−1^ azide stretch. ^13^C-NMR (75 MHz, CDCl_3_): δ 135.71, 134.34, 125.79, 122.40, 119.26, 116.19, 28.42, 22.69. HRMS calcld 176.1062, found 176.1058.


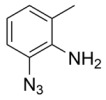


*2-azido-6-methylaniline*. 1 mmol of *o*-toluidine was added to a flame-dried Schlenk tube containing 0.10 mmol of CuBr. Then, trimethyl silyl azide (2 mmol) was added, followed by addition of 4 mL of freshly distilled acetonitrile. Finally, 2 mmol of TBHP (5.0–6.0 M in decane) was added, and the reaction was thermostatted at 30 °C for 6 h. After this, 15 mL of ethyl acetate was added, and the reaction mixture was concentrated on a rotary evaporator. This was then directly loaded onto a silica column and eluted with pet ether: ethylacetate (60:1). ^1^H-NMR (300 MHz, Chloroform-*d*): δ 6.94 (d, *J* = 7.9 Hz, 1H), 6.87 (d, *J* = 7.4 Hz, 1H), 6.73 (t, *J* = 7.7 Hz, 1H), 3.77 (s, 2H), 2.17 (s, 3H). IR: 2113 cm^−1^ azide stretch. ^13^C-NMR (75 MHz, CDCl_3_): δ 136.31, 126.74, 124.83, 123.46, 118.36, 115.96, 17.35. HRMS calcld 148.0749, 148.0746.


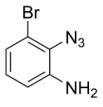


*2-azido-6-bromoaniline*. 2-Bromoaniline (1 mmol) was added to a flame-dried Schlenk tube containing 0.10 mmol of CuBr. Then, TMSN_3_ (2 mmol) was added, followed by addition of 4 mL of freshly distilled acetonitrile. Finally, 2 mmol of TBHP (5.0–6.0 M in decane) was added, and the reaction was heated to 30 °C for 6 h. Then, 15 mL of ethyl acetate was added, and the reaction mixture was evaporated. It was then directly loaded onto silica (hexane: ethylacetate (90:10)) to give the desired product in 23% isolated yield. ^1^H-NMR (400 MHz, Chloroform-*d*): δ 7.21 (dd, *J* = 8.0, 1.3 Hz, 1H), 6.98 (dd, *J* = 7.9, 1.3 Hz, 1H), 6.65 (t, *J* = 8.0 Hz, 1H), 4.25 (s, 2H). IR: 2117 cm^−1^ azide stretch. ^13^C-NMR (101 MHz, Chloroform-*d*): δ 136.51, 128.72, 125.97, 118.89, 117.31, 109.55. HRMS calcld 211.9697, found 211.9684.


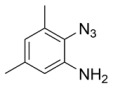


*2-azido-3,5-dimethylaniline*. 2,4 dimethyl aniline (1 mmol) was added to a flame-dried Schlenk tube contianing 0.1 mmol of CuBr. Then, TMSN_3_ (2 mmol) was added, followed by addition of 4 mL of freshly distilled acetonitrile. Finally, 2 mmol of TBHP (5.0–6.0 M in decane) was added, and the reaction was heated to 30 °C for 6 h. Then, 15 mL of ethyl acetate was added, and the reaction mixture was evaporated. It was then directly loaded on silica (Pet ether: EtOAc (60:1)) to give the desired product in 60% isolated yield. Analytical data matched literature [22].


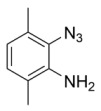


*2-azido-3,6-dimethylaniline*. 2,6-dimethyl aniline (1 mmol) was added to a flame-dried Schlenk tube containing 0.1 mmol of CuBr. Then, TMSN_3_ (2 mmol) was added, followed by addition of 4 mL of freshly distilled acetonitrile. Finally, 2 mmol of TBHP (5.0–6.0 M in decane) was added, and the reaction was heated to 30 °C for 6 h. Then, 15 mL of ethyl acetate was added, and the reaction mixture evaporated. It was then directly loaded onto silica (Pet ether: EtOAc (60:1)) to give the desired product in 80% isolated yield. ^1^H-NMR (300 MHz, Chloroform-*d*): δ 6.83 (d, *J* = 7.6 Hz, 1H), 6.52 (d, *J* = 7.6 Hz, 1H), 3.89 (s, 2H), 2.39 (s, 3H), 2.15 (s, 3H). ^13^C-NMR (75 MHz, Chloroform-*d*): δ 138.31, 130.58, 127.62, 123.77, 121.08, 120.15, 17.75, 17.32. HRMS calcld 162.0905, found 162.0900.


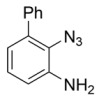


*3-azido-(1,1′-biphenyl)-2-amine*. 2-Phenylaniline (1 mmol) was added to a flame-dried Schlenk tube containing 0.10 mmol of CuBr. Then, TMSN_3_ (2 mmol) was added, followed by addition of 4 mL of freshly distilled acetonitrile. Finally, 2 mmol of TBHP (5.0–6.0 M in decane) was added, and the reaction was heated at 30 °C for 6 h. Then, 15 mL of ethyl acetate was added, and the reaction mixture evaporated. It was then directly loaded onto silica (Petroleum ether: ethylacetate (60:10)) to give the desired product in 60% isolated yield. Analytical data matched literature [22]. ^1^H-NMR (400 MHz, Chloroform-*d*): δ 7.48–7.34 (m, 5H), 7.05 (dd, *J* = 7.8, 1.5 Hz, 1H), 6.94 (dd, *J* = 7.6, 1.5 Hz, 1H), 6.85 (t, *J* = 7.7 Hz, 1H), 3.94 (s, 2H).


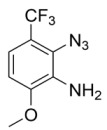


*2-azido-6-methoxy-3-(trifluoromethyl)aniline*. Using the same general procedure, isolated in 80% purity after eluting with 6:1 Hex:EtOAc. ^1^H-NMR (300 MHz, Chloroform-*d*) δ 7.02 (d, *J* = 8.7 Hz, 1H), 6.67 (d, *J* = 8.6 Hz, 1H), 4.19 (s, 2H), 3.90 (s, 3H). ^19^F-NMR (282 MHz, Chloroform-*d*): δ −59.44. HRMS calcld 232.0572, found 232.0545.

### 4.2. Catalytic Reactions to Give Azobenzenes

For the catalytic reactions, the following general procedure was followed: All reactions were carried out with 0.3 mmol of azide. **[Co^II^(TPP)]** (5 mol%) was transferred to a flame-dried Schlenk tube, after which the tube was evacuated and back-filled with dinitrogen three times. In a separate Schlenk tube containing 0.3 mmol of the azide, 4 mL of toluene was added to dissolve the azide. Using a syringe, this solution was transferred to a Schlenk tube containing the **[Co^II^(TPP)]** catalyst. The reaction mixture was then bubbled with dinitrogen for 15 min, after which it was heated to 90 °C for 18 h.

The reaction mixture was concentrated and was directly loaded onto a glass baked silica plate and ran using a suitable solvent (or solvent mixtures). The desired compound always gave a characteristic bright orange/red band on the silica plate.

**A**: *(E)-2,2′-(diazene-1,2-diyl)dianiline.* Using the general procedure (Prep-TLC using pure DCM), 80% isolated yield. Analytical data matched literature [21]. ^1^H-NMR (400 MHz, Chloroform-*d*): δ 7.68 (dd, *J* = 8.0, 1.6 Hz, 1H), 7.23–7.06 (m, 1H), 6.93–6.63 (m, 2H), 5.48 (s, 2H). ^13^C-NMR (126 MHz, CDCl_3_) δ 143.11, 137.73, 131.37, 124.29, 117.66, 117.04. HRMS calcld 211.1106, found 211.1106.

**C:*** (E)-6,6′-(diazene-1,2-diyl)bis(2-(tert-butyl)aniline).* Using the general procedure (Prep-TLC using DCM), 98% isolated yield. ^1^H-NMR (300 MHz, Chloroform-*d*): δ 7.50 (dd, *J* = 8.1, 1.4 Hz, 1H), 7.40–7.24 (d, 7.8 1H), 6.70 (t, *J* = 7.9 Hz, 1H), 1.49 (s, 9H). ^13^C-NMR (75 MHz, CDCl_3_): δ 145.00, 140.29, 135.73, 129.74, 117.76, 115.97, 35.10, 30.25. HRMS calcld 296.2001, found 296.2005.

**D:** *(E)-6,6′-(diazene-1,2-diyl)bis(2-isopropylaniline).* Using the general procedure (Prep-TLC using DCM), 98% isolated yield. ^1^H-NMR (300 MHz, Chloroform-*d*): δ 7.52 (dd, *J* = 8.1, 1.5 Hz, 1H), 7.19 (dd, *J* = 7.6, 1.4 Hz, 1H), 6.77 (t, *J* = 7.8 Hz, 1H), 5.24 (s, 2H), 3.10–2.83 (m, 1H), 1.32 (d, *J* = 6.8 Hz, 6H). ^13^C-NMR (75 MHz, CDCl_3_): δ 142.53, 138.65, 134.21, 117.61, 117.30, 27.67, 22.34. HRMS calcd. 325.2392 for C_20_H_28_N_4_, found 325.2394.

**E:** *(E)-3,3′-(diazene-1,2-diyl)bis(([1,1′-biphenyl]-2-amine)).* Using the general procedure (Prep-TLC using DCM: hexane = 1:1), 60% isolated yield. ^1^H-NMR (400 MHz, Chloroform-*d*): δ 7.70 (dd, *J* = 8.1, 1.6 Hz, 1H), 7.60–7.48 (m, 5H), 7.19 (dd, *J* = 7.2, 1.6 Hz, 1H), 6.86 (t, *J* = 7.7 Hz, 1H), 5.55 (s, 2H). ^13^C-NMR (101 MHz, Chloroform-*d*): δ 141.75, 138.80, 138.09, 132.64, 129.45, 129.35, 129.17, 129.11, 127.72, 121.42, 117.32. HRMS calcld 364.1688, found 364.1645.

**F**: *(E)-6,6′-(diazene-1,2-diyl)bis(2-bromoaniline)*. Using the general procedure (Prep-TLC using DCM: hexane = 1:1), 48% isolated yield. ^1^H-NMR (400 MHz, Chloroform-*d*): δ 7.64 (dd, *J* = 8.1, 1.5 Hz, 1H), 7.48 (dd, *J* = 7.8, 1.5 Hz, 1H), 6.70 (t, *J* = 7.9 Hz, 1H), 6.09 (s, 2H). HRMS calcld 367.9272, found 367.9256.

#### TD-DFT Calculations

The UV-Vis transitions of compound **F** were calculated with TD-DFT (nroots = 100; maxdim = 600; triplets = false), as implemented in the ORCA package at the b3-lyp level (RIJCOSX) using the def2-TZVP basis set [28,29,30,31]. We used COSMO [32,33,34,35,36] dielectric solvent corrections (ε = 8.93; CH_2_Cl_2_) to account for solvent effects.

## 5. Conclusions

In conclusion, we have reported a unique base metal-catalyzed dimerization reaction of substituted *o*-amino phenyl azides that is a general method for synthesizing *o*-amino-azobenzenes. Mechanistically, these reactions proceed via azide activation at the catalyst, leading to formation of porphyrin-Co(III)-*nitrene* radicals. These nitrene radical intermediates perform H-atom abstraction form the *ortho* amine substituent to form the **OPDI** reactive intermediates, which couple to form azobenzenes. This protocol is more efficient for azides with electron-donating substituents than those with electron-withdrawing substituents. The synthesized azobenzenes are bathochromically shifted compared to the unsubstituted azobenzenes. Based on the crystal structure, the *ortho*-amine substituent is seen to participate in H-bonding interactions with the azo N atom. This can be expected to cause hindered rotation for the *trans-* to *cis*- isomerization and thermal relaxation from *cis*- to *trans*- isomer can be expected to be fast. One of the possible applications of these *o*-amine substituted compounds may be as candidates for use as photo-responsive acids/bases. At the same time, the amine functionality in these compounds can act as a connection point for further functionalization.

## Figures and Tables

**Table 1:** Optimization of **[Co^II^(TPP)]**-catalyzed synthesis of azobenzene from azide **1** as a test substrate *.

Entry	Solvent	Temperature (°C)	Catalyst Loading	Yield
1	Toluene	90	5 mol%	98%
2	Benzene	60	5 mol%	-
3	Toluene	60	5 mol%	-
4	Toluene	90	1 mol%	60%
5	THF	60	5 mol%	-
6	Toluene	90	No catalyst	-

* All reactions were carried out with 0.3 mmol of azide, **[Co^II^(TPP)]** in 4 mL of toluene. The reaction mixtures were bubbled with dinitrogen for 15 min prior to thermostatting at the stated temperature for 18 h.

## Supplementary Materials

Supplementary materials are available on line.
