# Supplementary material for: Porphyrin Co(III)-Nitrene Radical Mediated Pathway for Synthesis of o-Aminoazobenzenes
Source: Molecules. 2018 May 1;23(5):1052. doi: 10.3390/molecules23051052 (PMC6102545; doi:10.3390/molecules23051052)
Supplement: Supplementary file 1 [file molecules-23-01052-s001.pdf]

Experimental data belonging to the paper

Porphyrin Co(III)-nitrene radical mediated pathway for synthesis of *o*-aminoazobenzenes

Monalisa Goswami[1] and Bas de Bruin[1,*]

[1] Homogeneous, Supramolecular and Bio-Inspired Catalysis, Van 't Hoff Institute for Molecular Sciences, University of Amsterdam, Amsterdam, the Netherlands

* Correspondence: B.deBruin@uva.nl

X-ray single crystal determination Compound C

$C_{20}H_{28}N_4$ Fw = 324.46, orange plate, 0.80 x 0.29 x 0.08 mm, monoclinic, P 21/c (no. 14), a = 11.7911(5), b = 6.7919(3), c = 11.5759(5)Å, α = 90 °, β = 94.530(2), γ = 90 °, V = 924.15(7) $Å^3$, Z = 2, D_x = 1.166 g cm^{-3}, μ = 0.071 mm^{-1}. In total, 7644 reflections were measured on a Bruker D8 Quest Eco diffractometer, equipped with a TRIUMPH monochromator and a CMOS PHOTON 50 detector (λ = 0.71073 Å) up to a resolution of $(\sin \theta/\lambda)_{max}$ = 0.84 $Å^{-1}$ at a temperature of 150(2) K. The intensity data were integrated with the Bruker APEX2 software.[1] Absorption correction and scaling was performed with SADABS.[2] (0.82–0.99 correction range). In total, 1615 reflections were unique (R_{int}= 0.110), of which 1280 were observed [$I>2\sigma(I)$]. The structure was solved with Intrinsic Phasing Methods using SHELXT[3] and refined with SHELXL-2013[4] against F^2 of all reflections. Non-hydrogen atoms were refined with anisotropic displacement parameters. Hydrogen atoms were introduced in calculated positions and refined with a riding model. R1/wR2 [$I > 2\sigma(I)$]: 0.0428/0.1482. S = 1.142. Residual electron density between -0.192 and 0.168 e $Å^{-3}$. Geometry calculations and checking for higher symmetry was performed with the PLATON program.[5]

[1] Bruker, APEX2 software, Madison, WI, USA, **2014**.

[2] SAINT, version 6.02, and SADABS, version 2.03; Bruker AXS, Inc., Madison, WI, **2002**.

[3] G.M. Sheldrick, SHELXT - Universität Göttingen, Germany, **2012**.

[4] G.M. Sheldrick, *Acta Cryst*. **2015**, A71, 3–8.

[5] A.L. Spek, *Acta Cryst*. **2009**, D65, 148–155.

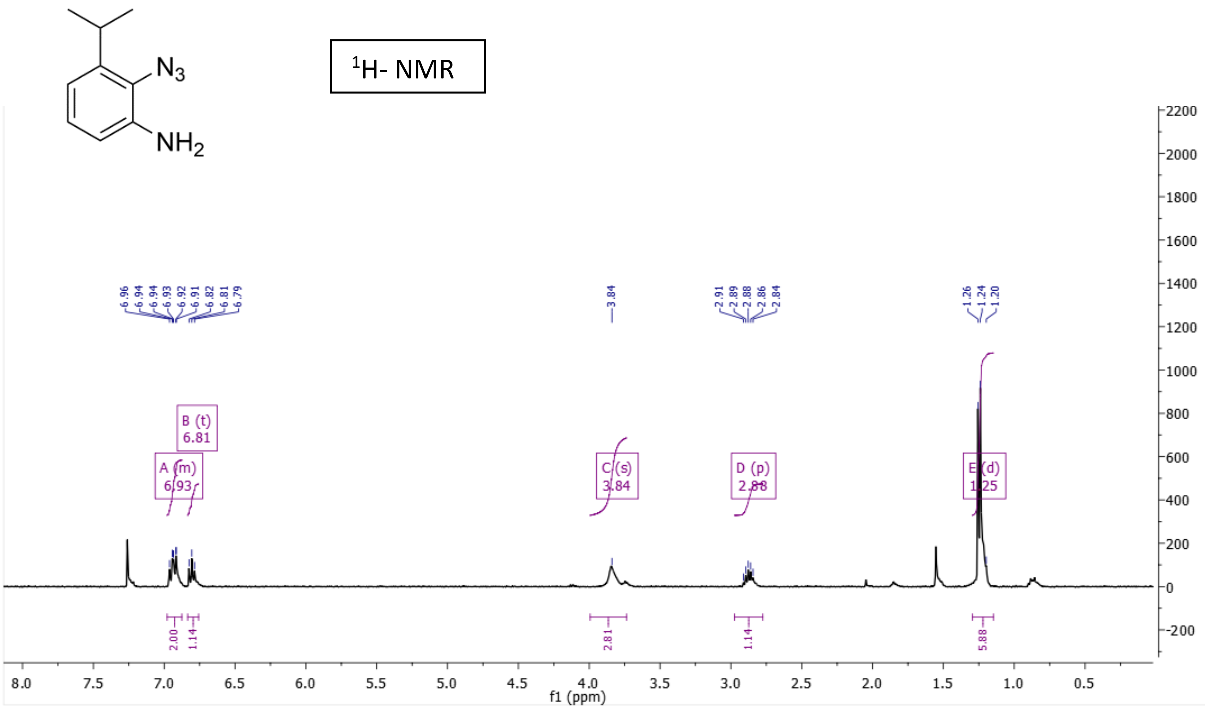
N3
NH2
1H- NMR
6.96 6.94 6.94 6.93 6.92 6.91 6.82 6.81 6.79
3.84
2.91 2.89 2.88 2.86 2.84
1.26 1.24 1.20
A (m) 6.93
B (t) 6.81
C (s) 3.84
D (p) 2.88
E (d) 1.25
2.00 1.14 2.81 1.14 5.88
f1 (ppm)

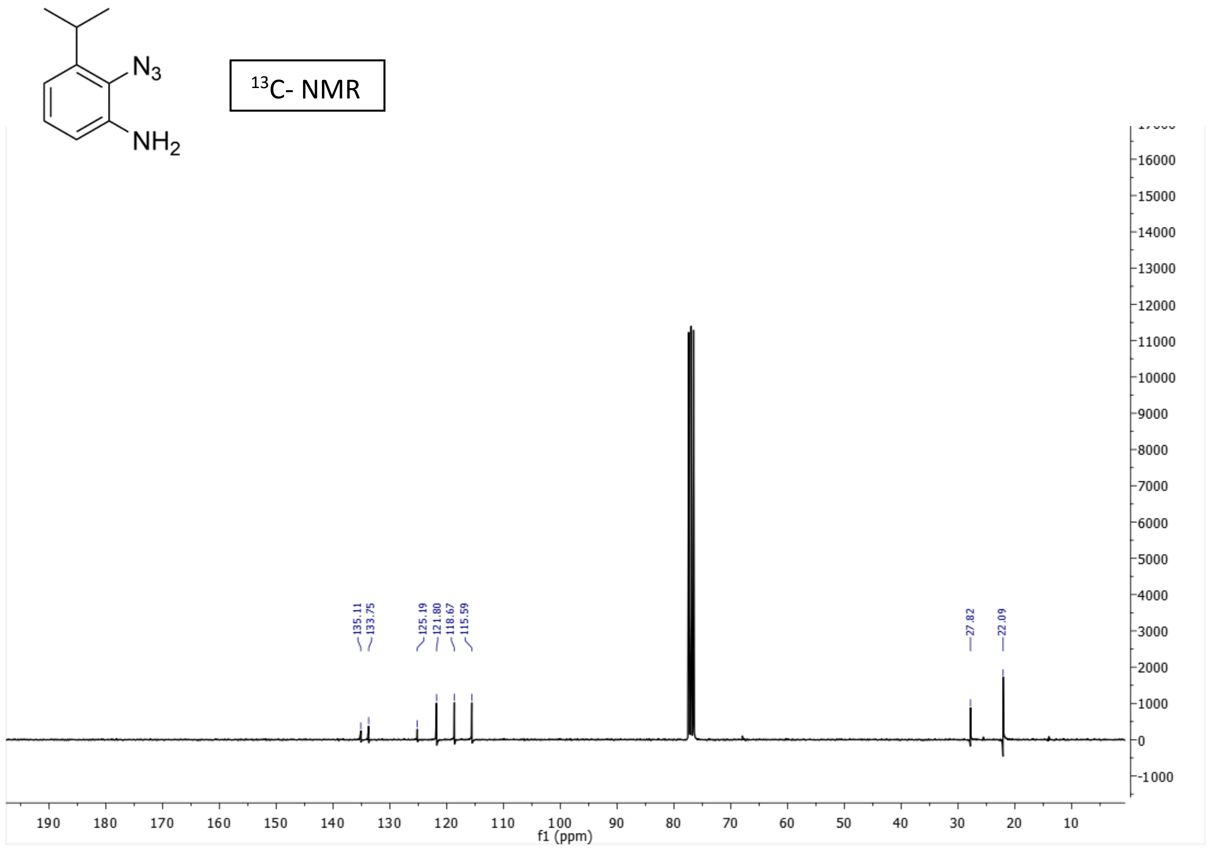
N3
NH2
13C- NMR
135.11 133.75 125.19 121.80 118.67 115.59
27.82 22.09
f1 (ppm)

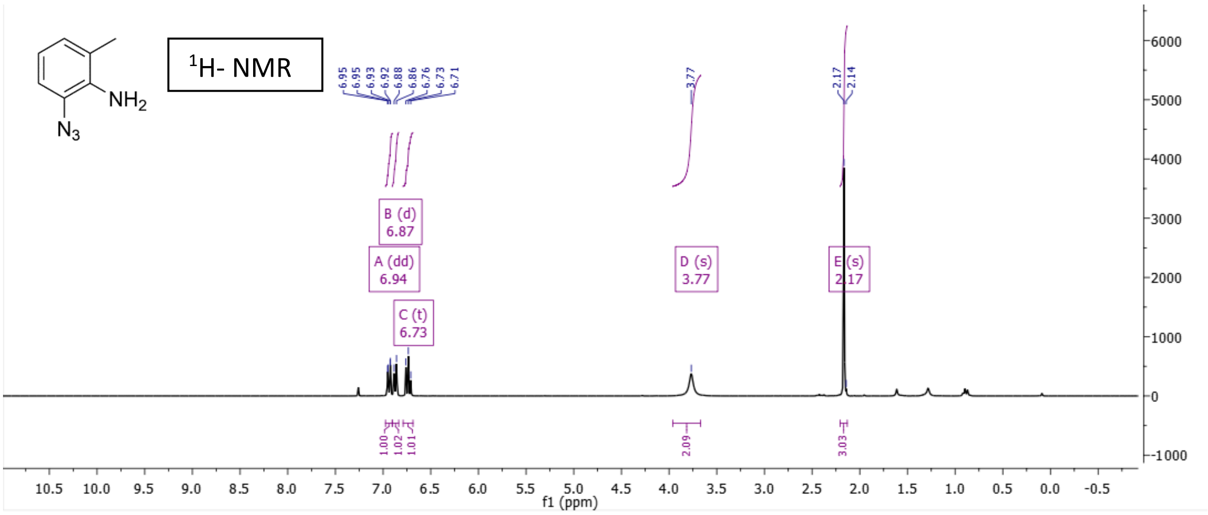
NH2
N3
1H- NMR
A (dd)
6.94
B (d)
6.87
C (t)
6.73
D (s)
3.77
E (s)
2.17
f1 (ppm)

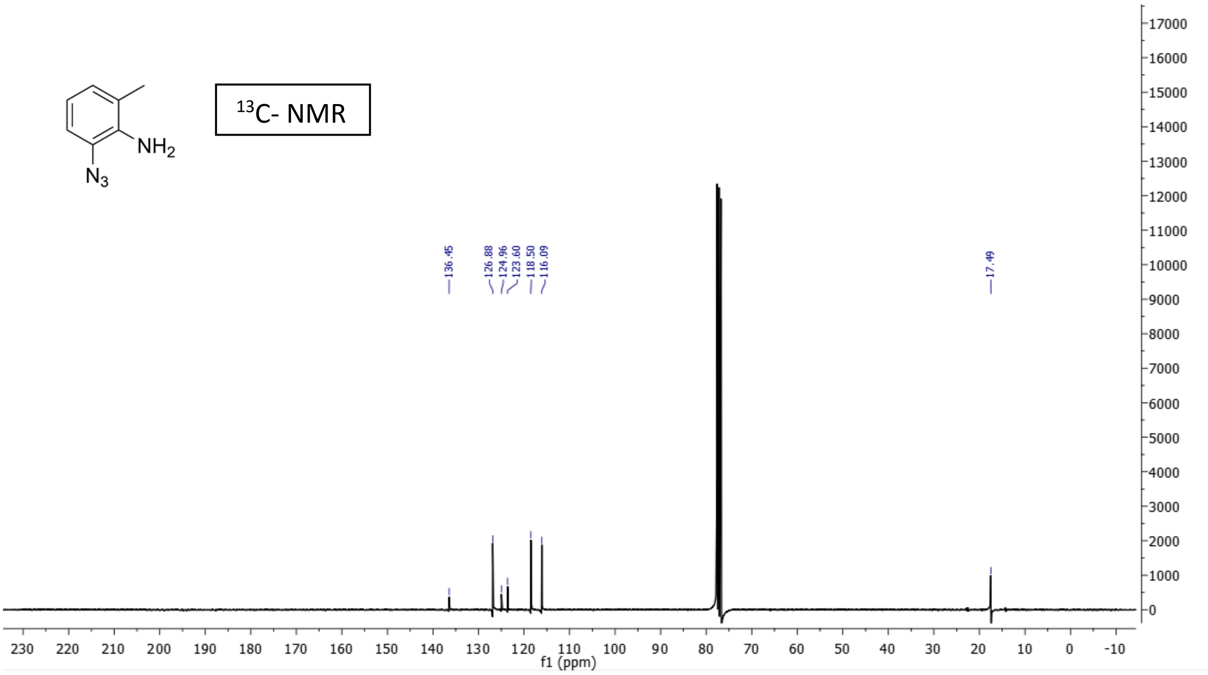
NH2
N3
13C- NMR
136.45
126.88
124.96
123.60
118.50
116.09
17.49
f1 (ppm)

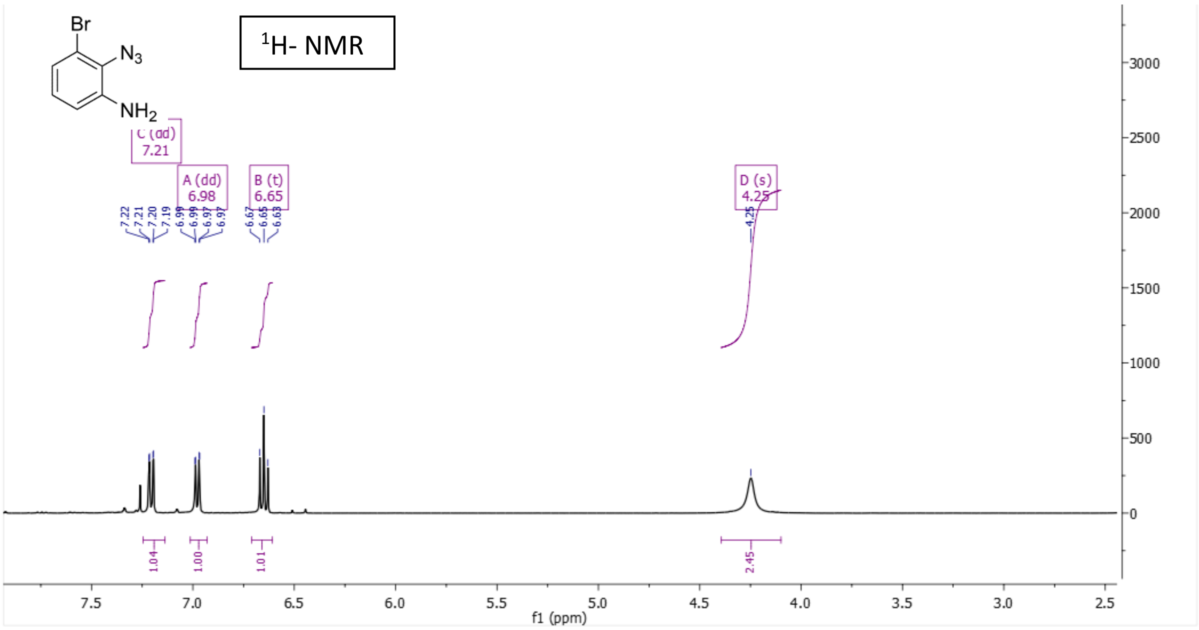

Br
N_3
NH_2
^{1}H- NMR
C (dd)
7.21
A (dd)
6.98
B (t)
6.65
D (s)
4.25
1.04
1.00
1.01
2.45
f1 (ppm)

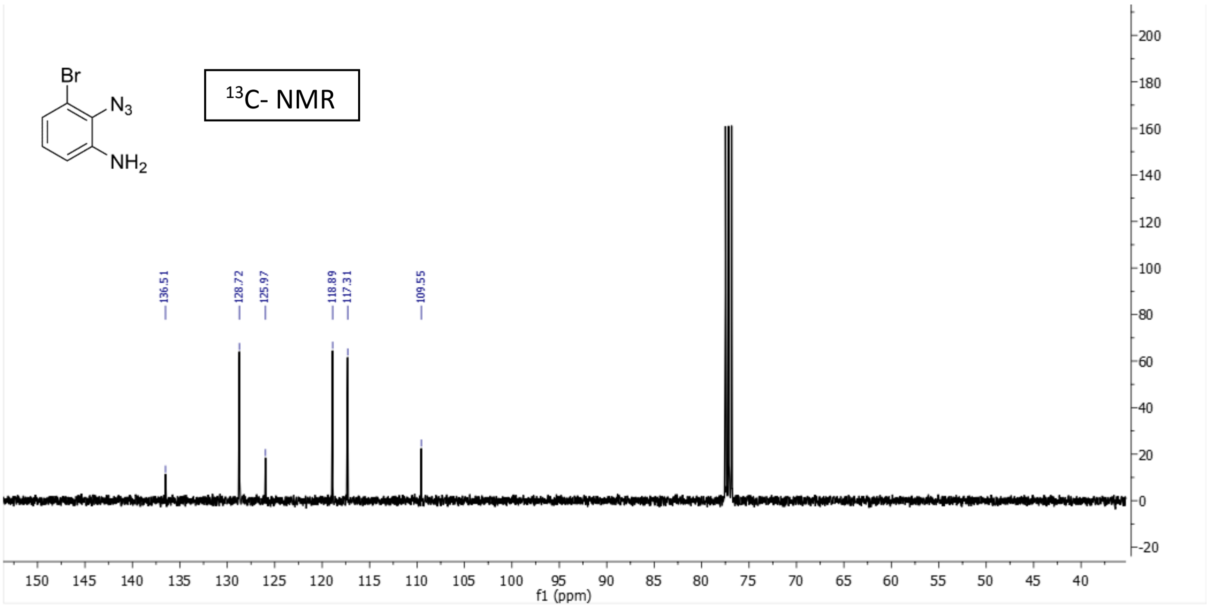

Br
N_3
NH_2
^{13}C- NMR
136.51
128.72
125.97
118.89
117.31
109.55
f1 (ppm)

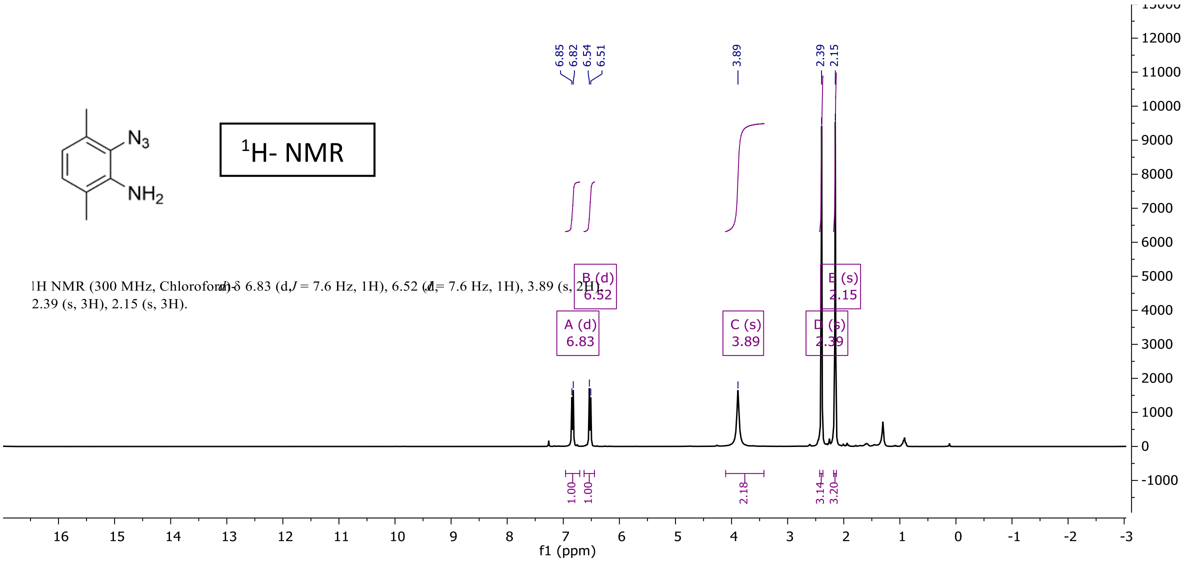

1H NMR (300 MHz, Chloroform-d) δ 6.83 (d, J = 7.6 Hz, 1H), 6.52 (d, J = 7.6 Hz, 1H), 3.89 (s, 2H), 2.39 (s, 3H), 2.15 (s, 3H).

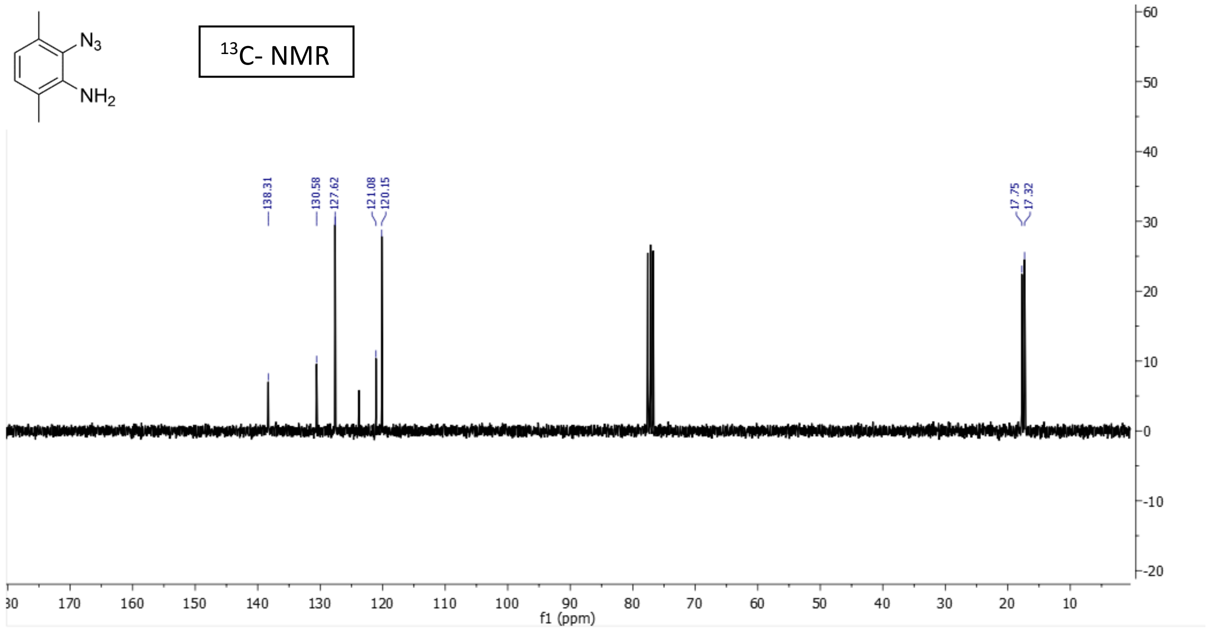

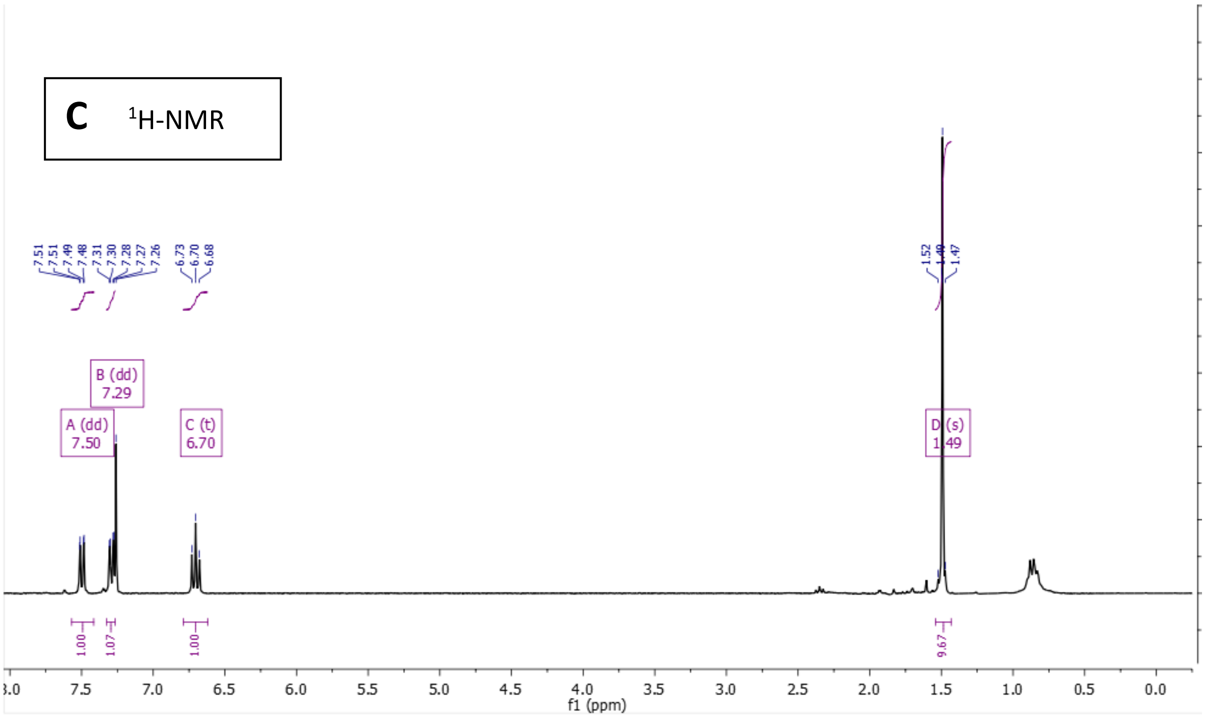
C
1H-NMR
A (dd)
7.50
B (dd)
7.29
C (t)
6.70
D (s)
1.49
f1 (ppm)

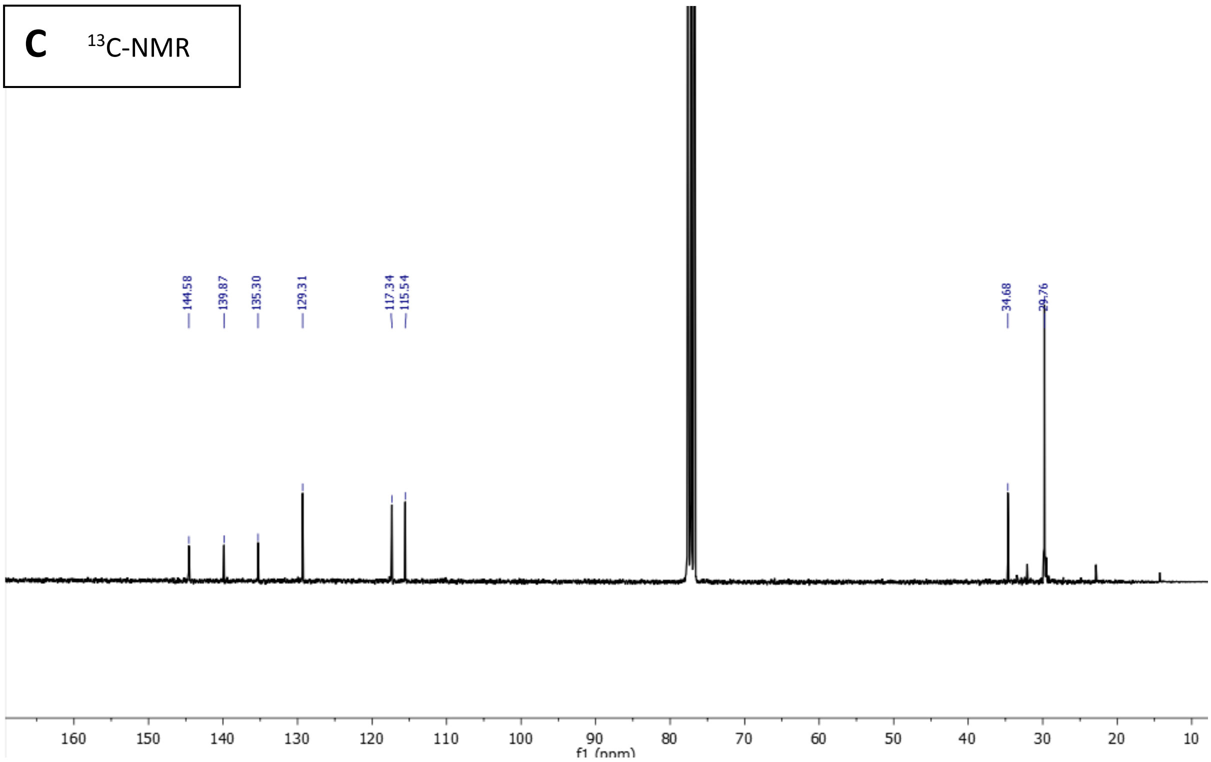
C
13C-NMR
144.58
139.87
135.30
129.31
117.34
115.54
34.68
29.76
f1 (ppm)

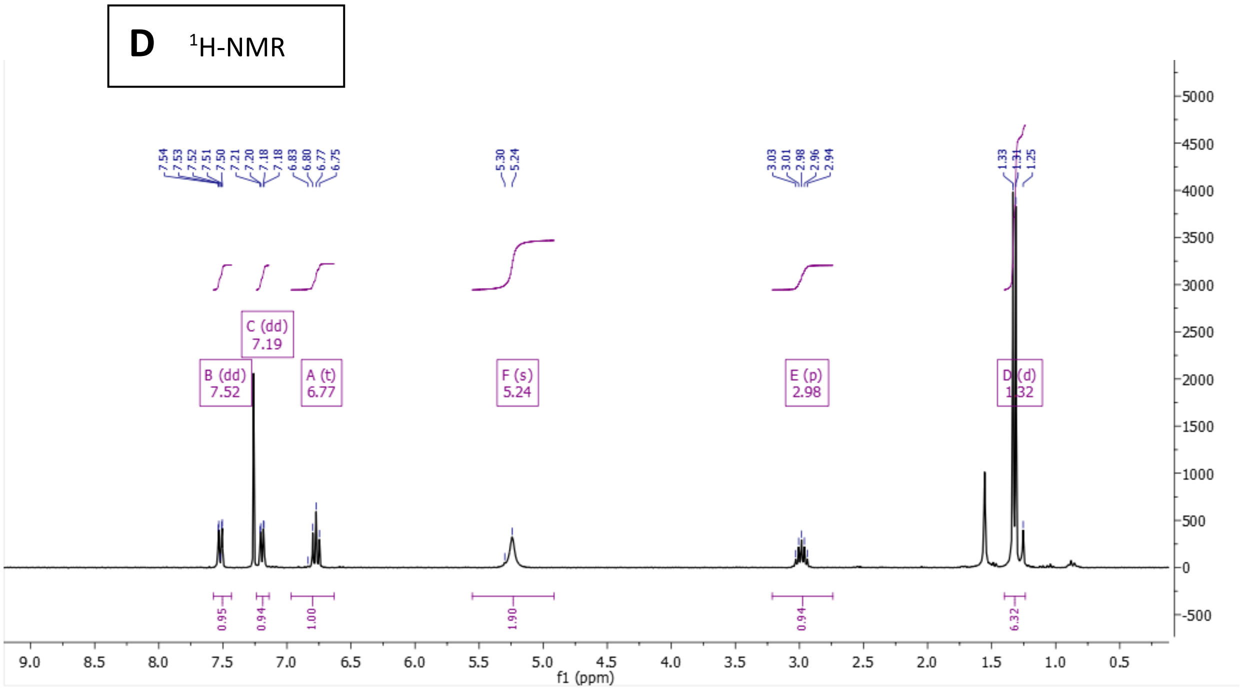
D
^{1}H-NMR
B (dd)
7.52
C (dd)
7.19
A (t)
6.77
F (s)
5.24
E (p)
2.98
D (d)
1.32
f1 (ppm)

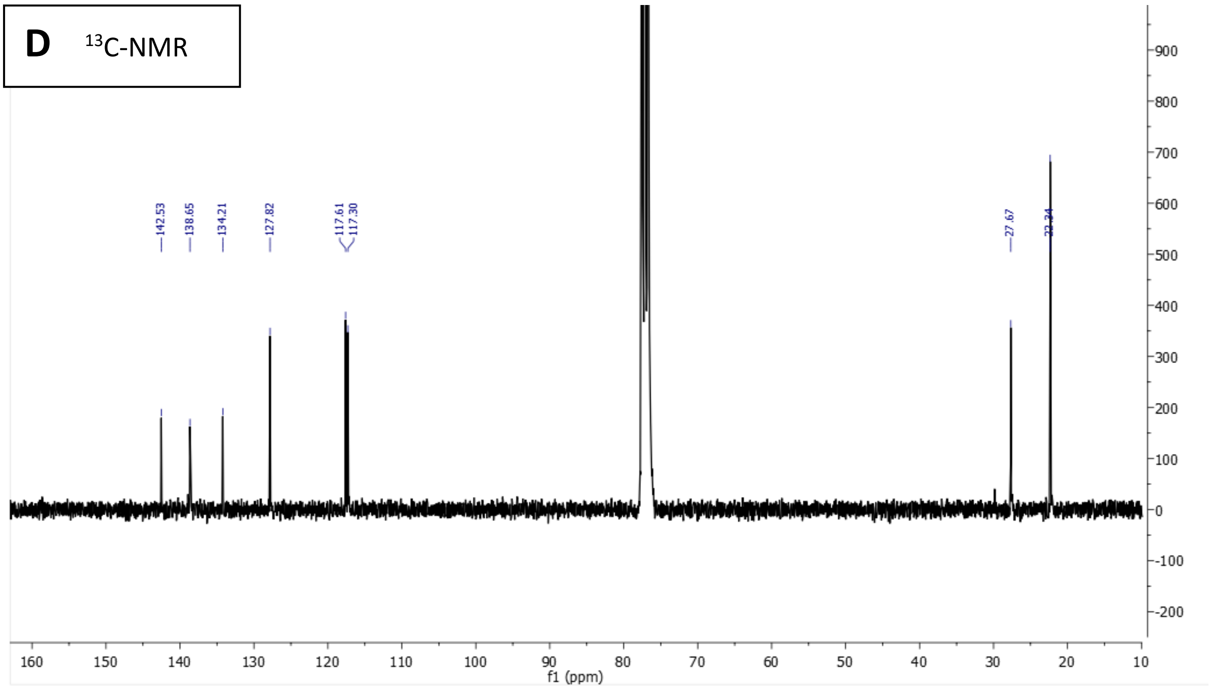
D
^{13}C-NMR
142.53
138.65
134.21
127.82
117.61
117.30
27.67
f1 (ppm)

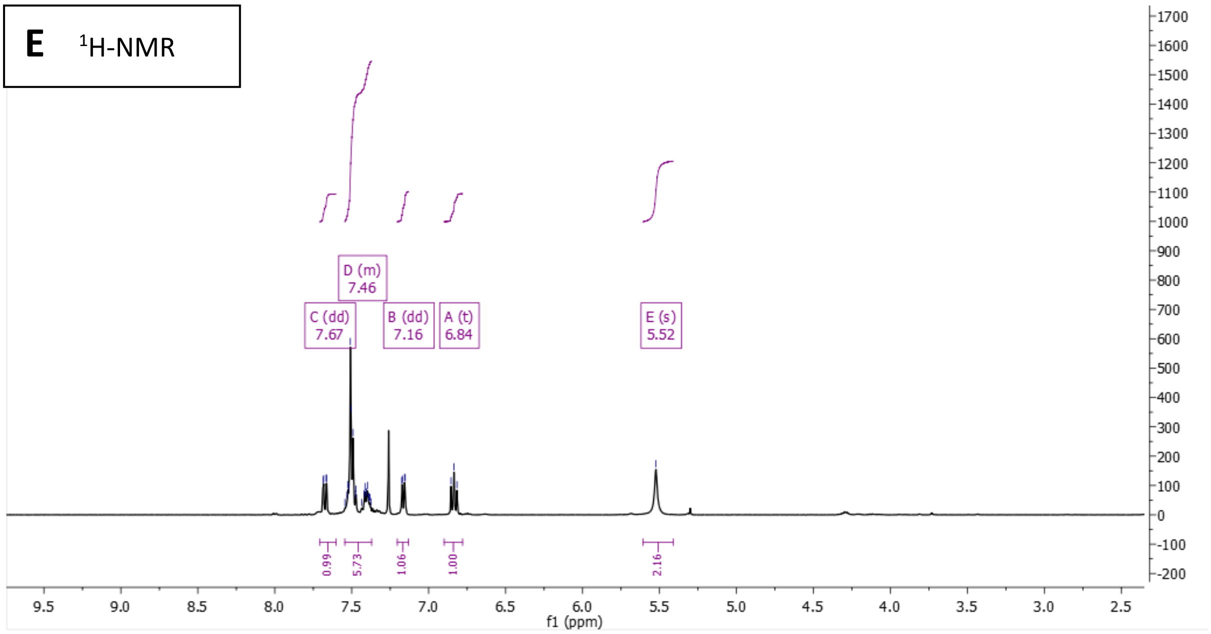
E
1H-NMR
C (dd)
7.67
D (m)
7.46
B (dd)
7.16
A (t)
6.84
E (s)
5.52
0.99
5.73
1.06
1.00
2.16
f1 (ppm)

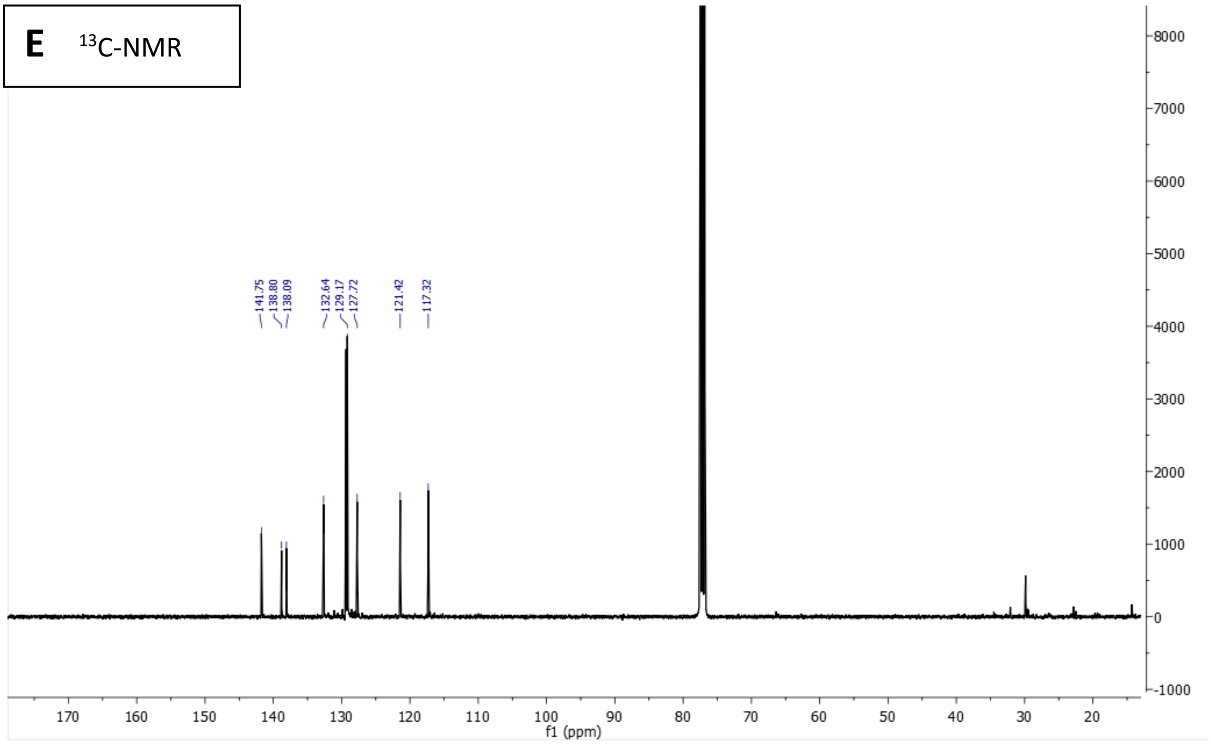
E
13C-NMR
141.75
138.80
138.09
132.64
129.17
127.72
121.42
117.32
f1 (ppm)

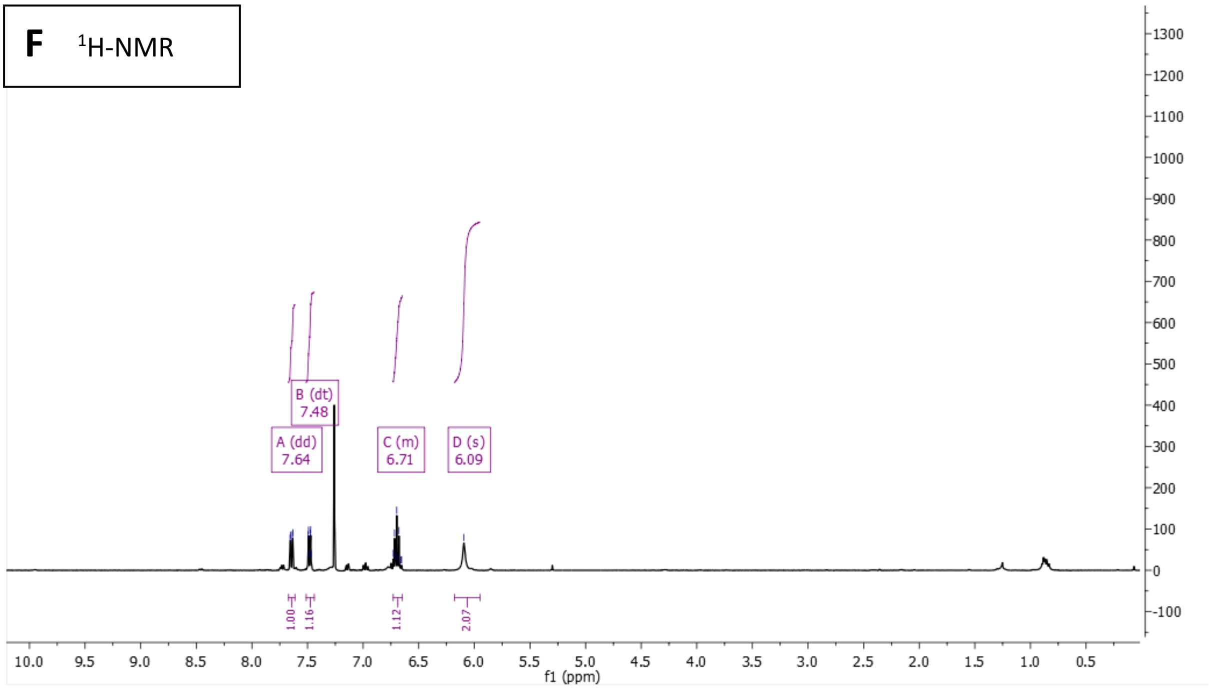
F
1H-NMR
A (dd)
7.64
B (dt)
7.48
C (m)
6.71
D (s)
6.09
1.00
1.16
1.12
2.07
f1 (ppm)
